# Comprehensive geriatric assessment and primary care based interventions for managing frailty in older adults: An evidence map

**DOI:** 10.1016/j.tjfa.2025.100104

**Published:** 2025-12-12

**Authors:** Smiteerekha Sahoo, Tanveer Rehman, Md Shaney Ali, Haimanti Bhattacharya, Kavitha AK, Rasmiranjan Nayak, Ashok Kumar Mahakuda, Sanghamitra Pati, Jaya Singh Kshatri

**Affiliations:** a ICMR- Regional Medical Research Centre, Bhubaneswar, Odisha, 751023, India; b Siksha 'O' Anusandhan Deemed to be University, Bhubaneswar, Odisha, India; c Indian Council of Medical Research, New Delhi, India

**Keywords:** Frailty, Older adults, Primary care, Evidence mapping, Low- and Middle-Income Countries (LMICs), Gap map

## Abstract

**Background:**

Frailty is a geriatric syndrome leading to adverse health outcomes, but can be managed through targeted interventions and potentially reversed. Primary care settings play a pivotal role in identifying and addressing frailty. This review aims to assess the effective primary care interventions and strategies to manage frailty.

**Methods:**

This review mapped evidence to evaluate systematic reviews of randomized controlled trials in older adults (≥60 years) on primary care-based interventions for managing frailty. Data were extracted from databases including MEDLINE, Embase, CINAHL, PsycINFO, and Cochrane CENTRAL, covering publications up to September 11, 2024. Interventions in primary care, community-based, or home-based settings were included, excluding hospitalized or bedridden individuals. The AMSTAR 2 tool assessed review quality, and interventions were categorized by type, setting, and effectiveness.

**Results:**

From the 3152 studies extracted, 17 systematic reviews met the inclusion criteria. Interventions were classified into physical, nutritional, pharmacological, e-health/telemedicine, and multicomponent approaches. Multicomponent interventions, combining physical, nutritional, and cognitive strategies, demonstrated effectiveness, with significant benefits reported in 15 reviews. Community and home-based settings dominated, emphasizing accessibility. However, the quality of evidence varied, with seven reviews rated as critically low and six as high. Most studies were conducted in high-income countries, limiting their generalizability to LMICs.

**Conclusion:**

Multicomponent interventions delivered in community settings show significant promise for managing frailty in older adults. However, evidence gaps suggest the need for context-specific research to adapt these interventions into primary care, which can improve the health status and quality of life for ageing populations globally.

## Highlights

• Multicomponent interventions were associated with higher effectiveness in managing frailty.
• Community and home-based interventions were found to be extensively used among different studies.
• Prominence of low rated evidence and lack of evidence from Low- and middle-Income countries (LMICs) was found.

## 1 Introduction

Frailty is a common condition in older adults, marked by heightened vulnerability and deterioration across several physiological systems [1]. This condition greatly affects the health, autonomy, and overall quality of life of older adults, frequently resulting in negative outcomes such as falls, hospital admissions, institutional care, and even death. Worldwide, frailty is responsible for nearly 19 % of the total Disability-Adjusted Life Years (DALYs) in people aged 70 and above, highlighting its significant role in the global disease burden [2].

The prevalence of frailty differs significantly across various regions and settings, impacting between 4.0 % and 59.1 % of older adults living in the community-dwelling older adult [3], with prevalence ranging from 19.0 % to 75.6 % among those in nursing homes or hospital settings [4]. The challenge is even greater in low- and middle-income countries (LMICs), where older populations face a significantly higher rate of frailty due to unequal healthcare access, socioeconomic inequalities, and the combined impact of communicable and non-communicable diseases [5,6]. In LMICs, frailty prevalence can reach as high as 35.9 % % in certain age groups, compared to 9.9 −13.6 % % in high-income countries (HICs) [7]. This disparity poses significant challenges for healthcare systems in LMICs, where resources are already stretched thin [8].

Although frailty is a serious condition, it is not an unavoidable outcome of aging. With appropriate and timely interventions, frailty can be delayed, reduced, or even reversed. Among the most effective strategies are physical exercise and nutritional support, including protein-enriched diets [9,10]. Physical exercise, particularly resistance and strength training, improves muscle mass, physical performance, and mobility. Nutritional programs, particularly those targeting malnourished older adults, have demonstrated improvements in functional outcomes and quality of life [11]. A multi-dimensional approach, incorporating medical, psychological, and social assessments, allows for the identification and management of frailty in a comprehensive and organized manner [12]. Studies report a 22 % improvement in functional independence among older adults undergoing Comprehensive Geriatric Assessment-based interventions [13]. Addressing social isolation and mental health through community engagement programs and counselling services has shown promising results in enhancing overall well-being and resilience among older adults. However, the effectiveness of these interventions varies by population and context, underscoring the need for tailored approaches. A holistic strategy combining physical, nutritional, and social interventions has demonstrated the greatest potential in managing frailty [14,15].

Primary care is ideally placed to tackle frailty, as it serves as the initial point of contact for the majority of older adults within the healthcare system. Using validated screening tools such as the Fried Phenotype Scale, Frailty Index, FRAIL scale, Edmonton Frailty Scale, and Clinical Frailty Scale, frailty can be identified early [16]. By integrating physical, nutritional, and psychosocial interventions into routine care, primary care practices can provide personalized and accessible solutions to manage frailty effectively [17]. Primary care settings are ideal for implementing community-driven initiatives, particularly important in LMICs where access to specialized care is limited [18]. Embedding frailty management into routine primary care practices enhances accessibility, implementation, and sustainability [[19], [20], [21]]. Despite its potential, significant gaps remain in the adoption and implementation of frailty interventions within primary care settings [22]. Fewer than a majority of primary care providers routinely screen for frailty, and even fewer incorporate tailored interventions into practice [16]. Therefore, our goal was to address these gaps by identifying and assessing primary care-based interventions for managing frailty in older adults aged 60 and above. We systematically reviewed existing interventions and summarized their effectiveness in enhancing both clinical outcomes and patient-reported experiences.

## 2 Materials and methods

### 2.1 Protocol registration

This protocol of this study was preregistered on the Open Science Framework (OSF) and is under an embargo to prevent scooping. It will be made public upon acceptance or publication. A view-only link will be made available with the journal editor to facilitate peer review and collaboration.

### 2.2 Study design

Following the framework of evidence gap maps, we constructed an evidence map that integrates user-friendly visual figures, interactive graphs, and searchable databases to enhance the utility of the findings. Similar approaches have been used in health by the International Initiative for Impact Evaluation (3ie), which has developed several EGMs focusing on interventions such as anaemia programs in low- and middle-income countries and performance measurement and management in primary care delivery systems [23].

### 2.3 Eligibility criteria

We included systematic reviews (with or without meta-analysis) that focused solely on Randomized Controlled Trials involving older adults (aged 60 years or older). Interventions implemented in primary care, community-based, or home-based settings, aimed at addressing frailty and measuring outcomes related to its reduction or improvement, were considered. Studies involving hospitalized or bedridden individuals were excluded, as were reviews that focused on specific patient groups, such as surgical patients, oncology cases, or those receiving palliative care.

### 2.4 Search strategy

A comprehensive literature search was performed in the following databases: MEDLINE (via PubMed), Embase, CINAHL, PsycINFO, and the Cochrane CENTRAL library. We utilized various search terms related to "Frailty" and "Systematic review" / "Meta-analysis" alongside appropriate headings. The search was conducted to include articles published up until September 11, 2024. Detailed search strategies for each database are provided as supplementary material. (Supplementary File 1)

### 2.5 Study selection

Following a calibration exercise, two reviewers (RN, AM) independently screened the search results to identify potentially eligible records based on title and abstract. Then, two reviewers (HB, AK) independently verified eligibility by reviewing the full-text articles of the selected records. In the event of disagreements, consensus was achieved through discussion or by involving a third reviewer (JSK). The article selection process was managed using Covidence© (www.covidence.org). Throughout the screening process, a team of experienced researchers (JSK, TR) ensured consistency among reviewers and offered assistance as needed.

### 2.6 Data extraction

Data was systematically extracted using a pre-designed spreadsheet to capture key details of each review, including characteristics of included systematic reviews (e.g., population demographics, geographical location), study settings (e.g., primary care, community-based, or home-based), types of interventions (e.g., physical, nutritional, pharmacological, e-health, or multi-component) and measured outcomes (e.g., frailty reduction, physical function), with comparators, effect sizes, and statistical significance of outcomes. The completed data extraction forms are available as supplementary files.

### 2.7 Assessment of the quality of included reviews

The methodological quality of the systematic reviews was evaluated using the AMSTAR 2 checklist, which emphasizes seven critical domains (listed below) that can significantly impact the validity of a review and its conclusions, along with nine noncritical domains [21].

• Protocol registered prior to the commencement of the review (item 2)
• Adequacy of the literature search (item 4)
• Justification for excluding individual studies (item 7)
• Risk of bias from studies included in the review (item 9)
• Appropriateness of meta-analytic methods (item 11)
• Consideration of risk of bias when interpreting the review results (item 13)
• Assessment of the presence and potential impact of publication bias (item 15)

Based on these criteria, the study quality in the results of each review was rated as high, moderate, low, or critically low [21]. A "high" rating indicated that there was no or only one non-critical weakness, providing a thorough and accurate summary of the available studies. A "moderate" rating was given when there were multiple non-critical weaknesses but no critical flaws, resulting in a reasonably accurate summary. Reviews with one critical flaw, regardless of the presence of non-critical weaknesses, were rated as "low," suggesting potential concerns regarding reliability [21]. Reviews with more than one critical flaw were deemed to have "critically low" study quality, making them unreliable for accurate synthesis of the evidence.

### 2.8 Evidence map

The evidence map categorized interventions based on:

• Setting:
  ○ Primary Care or Institutional Settings: Formal healthcare institutions such as clinics or hospitals.
  ○ Community or Home-Based Settings: Interventions delivered outside institutional settings.
• Outcomes:
  ○ No Effect: Interventions that did not demonstrate a statistically significant impact on frailty outcomes
  ○ Benefit: Interventions that yielded significant improvements in frailty-related outcomes.

The map used intuitive icons to represent the types of interventions, including physical (e.g., exercise programs), nutritional (e.g., protein supplementation), pharmacological, E-Health or Telemedicine and multicomponent interventions.

## 3 Results

**Fig. 1:**
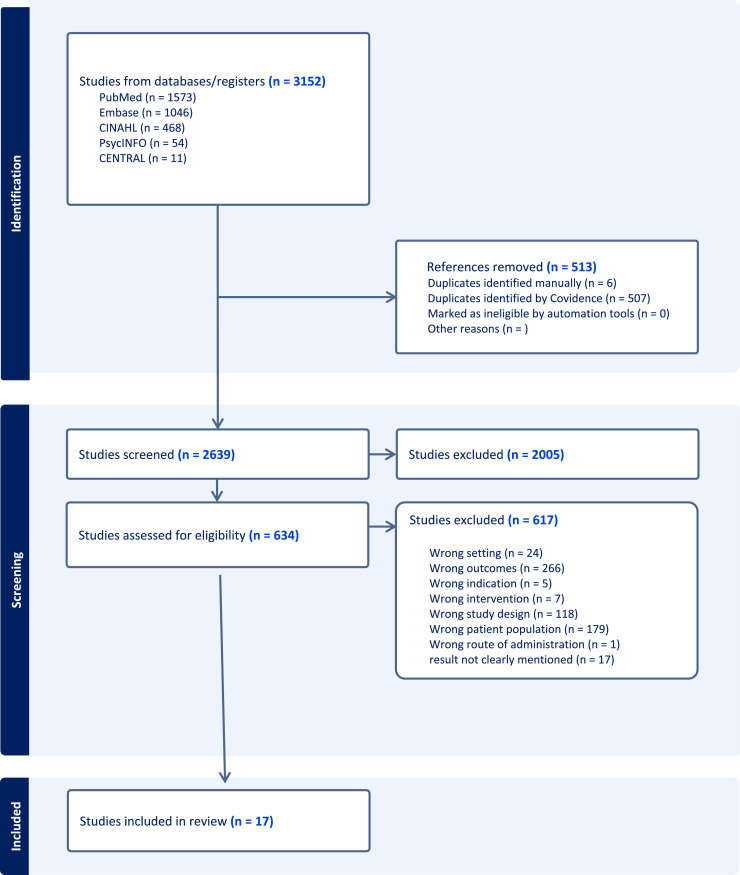
PRISMA diagram for evidence gap map synthesis.

The database searches yielded a total of 3152 studies. After deduplication and title/abstract screening, 634 studies underwent full-text evaluation, leading to the exclusion of 617 studies for various reasons, as depicted in the PRISMA flow chart (Fig. 1). In the end, 17 studies fulfilled all eligibility criteria and were included in the final analysis.

Table 1 outlines the characteristics of the included systematic reviews. There were six high-rated reviews based on the AMSTAR 2 critical domains checklist [[24], [25], [26], [27], [28], [29]]. There was one moderate-rated review that exhibited non-critical weaknesses, such as limited reporting detail on funding and heterogeneity of results of studies included, but it was still considered reasonably reliable [30]. Moreover, three Low-rated reviews presented critical flaws, potentially compromising the accuracy and comprehensiveness of their findings [[31], [32], [33]]. The remaining seven reviews were critically low-rated and possessed multiple critical weaknesses, indicating they should not be relied upon for decision-making [19,[34], [35], [36], [37], [38], [39]]. Further investigation into the specific items contributing to these ratings is warranted to understand each review's strengths and weaknesses fully. The reviews consisted of trials ranging from 4 included studies by Han and colleagues to 56 included studies by Sun and team [25,29]. Seven of these reviews conducted meta-analyses. The evidence reviewed was relatively recent, with search years spanning from 2015 to 2023. Most studies were conducted in HICs, reflecting a geographical bias in the available research.

**Table 1:** Primary care-based interventions for managing frailty among older adults.

Sl. No.	Study ID	Studies Included	Participants	Meta-Analysis	Year of Search	Countries	Study Quality
1	deLabra 2015	9	1067	No	2015	HICs; LMICs	Critically Low
2	Li 2022	13	3176	Yes	2021	HICs; LMICs	Low
3	Dedeyne 2017	8	1122	No	2016	HICs	High
4	Han 2023	4	205	No	2023	HICs	High
5	Macdonald 2020	31	4794	Yes	2019	HICs	High
6	Veninšek 2018	27	151	No	2017	HICs	Critically Low
7	Artaza-Artabe 2016	32	50,056	No	2016	HICs; LICs	Critically Low
8	Moraes 2021	19	1564	Yes	2019	HICs; LMICs	High
9	Kasa 2023	6	2297	No	2022	HICs	Low
10	Negm 2019	21	5262	Yes	2016	HICs	Critically Low
11	Esfandiari 2021	12	1819	Yes	2020	HICs	Critically Low
12	Daryanti Saragih 2022	15	1294	Yes	2021	HICs; LMICs	High
13	Wan 2022	12	1123	Yes	2022	HICs	Low
14	Travers 2019	46	15,690	No	2017	HICs; LMICs	Critically Low
15	Pazan 2021	25	4954	No	2019	HICs	Moderate
16	Sun 2023	56	9530	Yes	2021	HICs; LICs	High
17	deLabra 2015	21	5275	No	2015	HICs	Critically Low

***Abbreviations used*:** HMICs: High- and Middle-Income Countries; LICs: Low-Income Countries; LMICs: Low- and Middle-Income Countries.

### 3.1 Types of interventions

Table 2 provides an overview of the various interventions for frailty management, classified into five primary categories: physical, nutritional, pharmacological, e-health/telemedicine, and multicomponent approaches. Physical interventions (*n* = 4) primarily involved exercises designed to enhance muscle strength, balance, flexibility, and cardiovascular health. These interventions aimed to improve overall physical fitness and contribute to a reduction in frailty. Nutritional interventions (*n* = 1) focused on dietary supplementation, such as protein and vitamins, to address nutritional deficiencies and promote muscle strength, immune support, and general health—all critical components in managing frailty. Pharmacological interventions (*n* = 1) utilized medications or supplements to manage deficiencies, optimize health conditions, and address chronic illnesses that exacerbate frailty. E-health and telemedicine interventions (*n* = 2) leveraged digital tools, including mobile health applications, telemonitoring, telephone counselling, and video-guided home exercises. These digital approaches provided remote support and continuous care, making frailty management accessible even to those unable to attend in-person care. Lastly, multicomponent interventions (*n* = 9) combined various strategies, including physical exercises, nutritional support, cognitive and social engagement, comprehensive health assessments, and home safety modifications. These holistic approaches were the most extensively studied and demonstrated the most promising results for comprehensive frailty management.

**Table 2:** Summary of findings for frailty management.

Sl. No.	Study ID	Studies Included	Participants	Description of Intervention	Effectiveness on Frailty Reduction (findings of SR)	Outcome Measures	Meta-Analysis Summary
1	deLabra 2015	9	1067	Physical comprehensive training (leg extensions, hip flexions, double-arm pull downs, bicep curls).	No impact	Fried’s frailty criteria	N/A
2	Li 2022	13	3176	Physical activity, exercise, fitness.	Significant	CHS criteria	MD = −0.73 (95 % CI: −1.05 to −0.41)
3	Dedeyne 2017	8	1122	Multidomain interventions: exercise, nutrition (protein, vitamins, milk fat, advice), hormones, cognitive, or psychosocial interventions.	Significant	CHS criteria	N/A
4	Han 2023	4	205	E-health interventions: telephone counselling, home video exercise, telemonitoring, mobile health programs.	No impact	Fried Frailty Criteria	N/A
5	Macdonald 2020	31	4794	Exercise with/without nutrition supplementation or education, comprehensive geriatric assessment.	No impact	Fried Frailty Criteria	RR = 0.62 (95 % CI: 0.48 to 0.79)
6	Veninšek 2018	27	151	Multicomponent training: physical activity, vitamin D, home modification, cognitive training, nutrition.	Significant	Frailty Index	N/A
7	Artaza-Artabe 2016	32	50,056	Protein intake, nutritional supplementation, vitamin D, exercise.	No impact	Fried phenotype	N/A
8	Moraes 2021	19	1564	Nutritional education, protein/energy supplements, specific diets with other interventions.	Significant	CHS criteria	OR = 2.30 (95 % CrI: 0.72 to 7.01)
9	Kasa 2023	6	2297	Multicomponent intervention: face-to-face activities, telephone support, exercise, nutrition, cognitive training.	No impact	Frailty Index	N/A
10	Negm 2019	21	5262	Physical activity, nutrition supplementation, protein, comprehensive geriatric assessment.	Significant	Frailty Index	SMD = −0.92 (95 % CI: −1.55 to −0.29)
11	Esfandiari 2021	12	1819	Remote health programs: phone, smartphone, tablet, computer, DVD.	Significant	Fried theoretical framework	SMD = 0.31 (95 % CI: 0.15 to 0.47)
12	Daryanti Saragih 2022	15	1294	Resistance band exercises.	Significant	Fried phenotype score	SMD = −0.29 (95 % CI: −0.55 to −0.03)
13	Wan 2022	12	1123	Baduanjin (Chinese exercise): warmup, main exercise, stretching.	No impact	Fried phenotype score	SMD = −1.46 (95 % CI: −2.39 to −0.53)
14	Travers 2019	46	15,690	Physical activity, health education, nutrition, home visits, hormone supplementation, counselling.	No impact	Fried Frailty Criteria	N/A
15	Pazan 2021	25	4954	Pharmacological interventions or medication optimization.	Significant	Fried Frailty Criteria	N/A
16	Sun 2023	56	9530	Non-pharmacological interventions: mind-body exercise, mixed physical training, resistance, aerobic, cognitive training.	No impact	SUCRA criteria	SMD = 0.34 (95 % CI: 0.23–0.45)
17	Apóstolo 2018	21	5275	Home/group-based physical exercise, nutrition consultation, computerized balance training, protein-calorie/micronutrient supplementation.	No impact	CHS criteria	N/A

### 3.2 Effectiveness of interventions

The effectiveness of the interventions was evaluated based on their ability to reduce frailty, with outcomes classified as either beneficial or having no effect. Among the 17 studies reviewed, 15 reported significant benefits in reducing frailty, with multi-component interventions showing the most notable impact. Only two interventions demonstrated no significant effect, indicating the potential need for further optimization or more targeted implementation of specific approaches.

### 3.3 Settings of interventions

The interventions were delivered in two primary settings: primary care or institutional settings, and community or home-based settings. Primary care and institutional settings included formal healthcare environments such as clinics and hospitals. In contrast, community and home-based settings involved interventions implemented in familiar, everyday environments, which enabled personalized care and enhanced adherence. Most interventions were conducted in community or home-based settings, emphasizing the importance of accessible and convenient care integrated into daily living environments.

### 3.4 Gaps in evidence

**Fig. 2:**
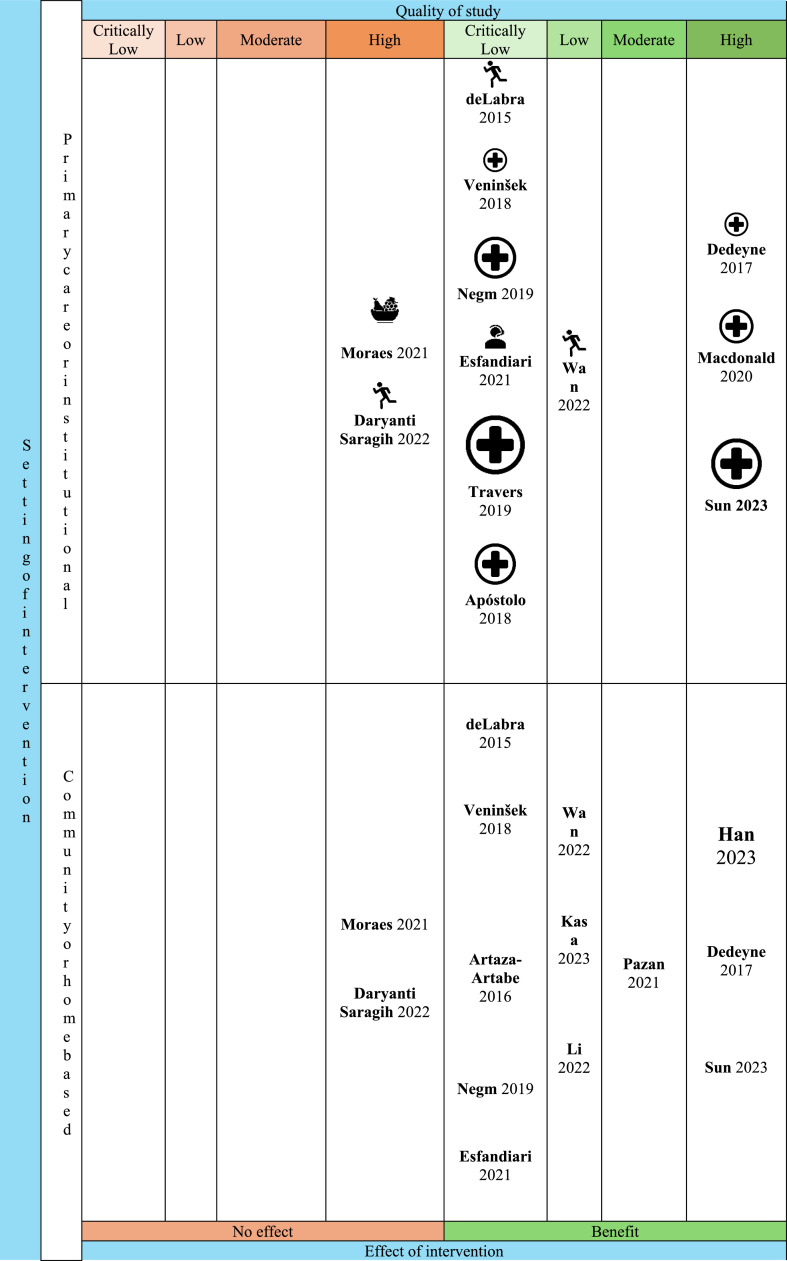
Distribution of intervention effects across settings and quality of study (AMSTAR-2). The vertical axis delineates the intervention setting as either "Primary care or institutional" (upper section) or "Community or home based" (lower section). The horizontal axis represents the "Effect of intervention," ranging from "No effect" (left, orange shaded region) to "Benefit" (right, green shaded region). Each study is positioned within a grid cell according to its quality rating (Critically Low, Low, Moderate, High) and observed effect. The study label, showing the first author and publication year, is displayed next to an icon representing the intervention type (Physical, Nutritional, Pharmacological, E-health, Multicomponent). Icon size reflects total participants in the study to visually indicate study weight. Icon interpretation:  Physical intervention  Nutritional intervention  Pharmacological Intervention  E-health/Telemedicine Intervention  Multicomponent Intervention.

The evidence map (Fig. 2) offers a comprehensive overview of interventions aimed at reducing frailty in older adults. It categorizes these interventions according to their type, setting, effectiveness, and the quality in the findings. While multi-component interventions consistently showed strong effectiveness, further evidence is especially needed to comprehend the impact of pharmacological and e-health interventions in LMIC settings.

### 3.5 Quality of evidence

The quality of the included systematic reviews was evaluated using the AMSTAR 2 tool, revealing considerable variability in methodological rigor. Six reviews were rated as high quality of study, indicating reliable evidence with no or only one non-critical weakness. Three reviews received a moderate quality of study rating, with more non-critical weaknesses but no critical flaws. One review was rated as low quality of study due to a critical flaw, raising concerns about its reliability. The remaining seven reviews were rated as critically low quality of study, highlighting multiple critical flaws and significant limitations in their methodological soundness.

## 4 Discussion

Our study systematically mapped evidence on primary care-based interventions for managing (reducing or improving) frailty among older adults, identifying five categories of interventions: physical, nutritional, pharmacological, e-health, and multicomponent approaches. Multicomponent interventions emerged as the most effective, combining strategies to address the multifactorial nature of frailty. Interventions delivered in community or home-based settings were prominent, emphasizing accessibility. The findings highlight significant evidence gaps, particularly in LMICs, and underscore variability in the quality of the evidence.

The effectiveness of multicomponent interventions, integrating physical, nutritional, and cognitive components, was a key finding. These interventions align with literature from high-income settings, where studies have consistently demonstrated their ability to improve mobility, muscle strength, and reduce frailty-related outcomes [40,41]. However, a notable challenge is disentangling the individual contributions of each component within multicomponent approaches, making it difficult to optimize specific strategies. In LMICs, barriers such as limited healthcare resources and infrastructure necessitate research on simplified yet effective models of multicomponent interventions to ensure feasibility and scalability.

Community and home-based interventions featured prominently across the studies, highlighting their effectiveness and potential for improving accessibility and adherence [42]. Consistent with similar studies, these interventions leverage familiar environments to foster participant engagement and ensure continuity of care [36,43]. However, in LMICs, where home-based care is constrained by resource limitations and a lack of trained personnel, the scalability of such interventions remains a concern. Addressing these gaps through capacity-building efforts and leveraging technology, such as telemedicine, can enhance the reach of frailty interventions in underserved settings.

Physical and nutritional interventions were widely studied and consistently shown to reduce frailty [44], as seen in Liu et al. (2022), where strength and balance exercises significantly improved mobility and reduced fall risks [45,46]. However, inconsistencies arose in LMIC contexts, where challenges such as inadequate funding, poor nutritional security, and limited access to exercise facilities hinder implementation [33]. This shows a ‘know-do’ gap and implementation research is needed to fill this and adapt these interventions to the local context. Health system and policy research promoting multicomponent, community-based interventions could improve health outcomes for aging populations, particularly in resource-constrained settings. Scaling these interventions through programs like telemedicine and home-based care can bridge the healthcare access gap in rural and underserved regions [38].

The quality of evidence varied considerably, with many studies being rated as critically low according to the AMSTAR 2 tool. This finding aligns with previous reviews highlighting a lack of standardized methodologies in frailty research. While high-quality studies strengthen the evidence for the effectiveness of interventions, the predominance of lower-quality studies highlights the need for more rigorous research designs, improved reporting standards, and systematic evaluations. Enhanced methodological rigor will enable more reliable synthesis and stronger policy recommendations.

A key strength of this study is its use of evidence mapping, offering a clear and visual representation of the intervention landscape for frailty in primary care and community settings. The systematic approach to data extraction and quality assessment ensures the reliability of identifying effective interventions. Only systematic reviews were included in the mapping, ensuring that evidence already synthesized was considered. The quality of the studies varied, with many being rated as critically low. Most of the studies were conducted in high-income countries, limiting their applicability to low- and middle-income countries (LMICs). Furthermore, the diversity of interventions resulted in variability in outcomes.

Future research should focus on developing context-specific interventions for LMICs, with an emphasis on scalability and sustainability. Standardizing frailty definitions and outcome measures will enhance comparability and support more robust meta-analyses. Additionally, leveraging digital health innovations, such as telemedicine and mobile health applications, presents promising opportunities to expand access to frailty care, particularly in resource-constrained settings. Policymakers and health systems must prioritize the integration of evidence-based frailty interventions into primary care frameworks, as this approach has the potential to significantly reduce the burden of frailty and improve the quality of life for aging populations worldwide.

## 5 Conclusion

Multicomponent interventions combined with community-based care have proven effective in reducing frailty in high-income countries (HICs). Future initiatives should focus on adapting and scaling evidence-based multicomponent interventions in resource-limited settings to improve frailty outcomes and enhance the quality of life for older adults worldwide.

## Funding

Multi Frame- funding: Intramural of ICMR.

## Author contributions

The concept and design were handled by S.P., S.S., J.S.K., and T.R.: S.P., S.S., J.S.K., and T.R. monitored analysis and critical revision of the manuscript for important intellectual content; H.B, KAK., A.M., and M.S.A,R.N. were responsible for conceptualization, formal analysis, methodology, writing original draft preparation.

## Ethical approval

### Institutional review board statement

Multi Frame IEC number: Reference *ICMR/IHEC-2024/015,* Date:13/06/2024.

## Data availability statement

All data from the study are available as an additional file.

## CRediT authorship contribution statement

**Smiteerekha Sahoo:** Writing – original draft, Validation, Investigation, Formal analysis, Data curation. **Tanveer Rehman:** Writing – review & editing, Writing – original draft, Validation, Software, Methodology, Investigation, Conceptualization. **Md Shaney Ali:** Writing – review & editing, Validation. **Haimanti Bhattacharya:** Writing – review & editing, Investigation, Formal analysis. **Kavitha AK:** Resources, Methodology, Investigation, Data curation. **Rasmiranjan Nayak:** Validation, Methodology, Investigation. **Ashok Kumar Mahakuda:** Validation, Methodology, Investigation. **Sanghamitra Pati:** Writing – review & editing, Supervision, Conceptualization. **Jaya Singh Kshatri:** Writing – review & editing, Supervision, Resources, Methodology, Conceptualization.

## Declaration of competing interest

The authors declare the following financial interests/personal relationships which may be considered as potential competing interests:

Jaya Singh Kshatri reports financial support was provided by Indian Council of Medical Research. If there are other authors, they declare that they have no known competing financial interests or personal relationships that could have appeared to influence the work reported in this paper.

## Appendix Supplementary materials

Image, application 1

Image, application 2

## References

[1] Fried L.P., Ferrucci L., Darer J., Williamson J.D., Anderson G. (2004). Untangling the concepts of disability, frailty, and comorbidity: implications for improved targeting and care. J Gerontol A Biol Sci Med Sci.

[2] Beard J.R., Officer A., Carvalho I.A. (2016). The World report on ageing and health: a policy framework for healthy ageing. Lancet.

[3] Collard R.M., Boter H., Schoevers R.A., Oude Voshaar R.C. (2012). Prevalence of frailty in community-dwelling older persons: a systematic review. J Am Geriatr Soc.

[4] Liu W., Puts M., Jiang F., Zhou C., Tang S., Chen S. (2020). Physical frailty and its associated factors among elderly nursing home residents in China. BMC Geriatr.

[5] Fried L.P., Tangen C.M., Walston J. (2001). Frailty in older adults: evidence for a phenotype. J Gerontol A Biol Sci Med Sci.

[6] Seligman B., Agarwal A., Bloom D.E. (2024). Frailty among older Indians: state-level factors. Popul Ageing.

[7] Melo R.C., Cipolli G.C., Buarque G.L.A. (2020). Prevalence of frailty in Brazilian older adults: a systematic review and meta-analysis. J Nutr Health Aging.

[8] Clegg A., Young J., Iliffe S., Rikkert M.O., Rockwood K. (2013). Frailty in elderly people. Lancet.

[9] Aguirre L.E., Villareal D.T. (2015). Physical exercise as therapy for frailty. Nestle Nutr Inst Workshop Ser.

[10] Morley J.E., Argiles J.M., Evans W.J. (2010). Nutritional recommendations for the management of sarcopenia. J Am Med Dir Assoc.

[11] Chavarro-Carvajal D.A., Ayala A.M., Venegas-Sanabria L.C. (2022). Use of a nutrition-focused quality improvement program for community-living older adults at malnutrition risk is associated with better nutritional outcomes. Clin Nutr ESPEN.

[12] Wleklik M., Uchmanowicz I., Jankowska E.A. (2020). Multidimensional approach to frailty. Front Psychol.

[13] Giri S., Barlow B., Al-Obaidi M. (2021). Trajectories of functional status among older adults receiving treatment for gastrointestinal (GI) malignancies: a report from the CARE study. J Clin Oncol.

[14] Hoogendijk E.O., van Hout H.P.J., Heymans M.W. (2014). Explaining the association between educational level and frailty in older adults: results from a 13-year longitudinal study in the Netherlands. Ann Epidemiol.

[15] Kojima G., Liljas A.E.M., Iliffe S. (2019). Frailty syndrome: implications and challenges for health care policy. Risk Manag Healthc Policy.

[16] Walston J., Buta B., Xue Q.L. (2018). Frailty screening and interventions: considerations for clinical practice. Clin Geriatr Med.

[17] Abbasi M., Rolfson D., Khera A.S., Dabravolskaj J., Dent E., Xia L. (2018). Identification and management of frailty in the primary care setting. CMAJ.

[18] Sriram V., Bennett S. (2020). Strengthening medical specialisation policy in low-income and middle-income countries. BMJ Glob Health.

[19] Travers J., Romero-Ortuno R., Bailey J., Cooney M.T. (2019). Delaying and reversing frailty: a systematic review of primary care interventions. Br J Gen Pract.

[20] Miake-Lye I.M., Hempel S., Shanman R., Shekelle P.G. (2016). What is an evidence map? A systematic review of published evidence maps and their definitions, methods, and products. Syst Rev.

[21] Shea B.J., Reeves B.C., Wells G. (2017). AMSTAR 2: a critical appraisal tool for systematic reviews that include randomised or non-randomised studies of healthcare interventions, or both. BMJ.

[22] Lacas A., Rockwood K. (2012). Frailty in primary care: a review of its conceptualization and implications for practice. BMC Med.

[23] Snilstveit B., Vojtkova M., Bhavsar A., Stevenson J., Gaarder M. (2016). Evidence & Gap Maps: a tool for promoting evidence informed policy and strategic research agendas. J Clin Epidemiol.

[24] Dedeyne L., Deschodt M., Verschueren S., Tournoy J., Gielen E. (2017). Effects of multi-domain interventions in (pre)frail elderly on frailty, functional, and cognitive status: a systematic review. Clin Interv Aging.

[25] Han H.W., Park S.W., Kim D.Y. (2023). E-health interventions for older adults with frailty: a systematic review. Ann Rehabil Med.

[26] Macdonald S.H.F., Travers J., Shé ÉN (2020). Primary care interventions to address physical frailty among community-dwelling adults aged 60 years or older: a meta-analysis. PLoS One.

[27] Moraes M.B., Avgerinou C., Fukushima F.B., Vidal E.I.O. (2021). Nutritional interventions for the management of frailty in older adults: systematic review and meta-analysis of randomized clinical trials. Nutr Rev.

[28] Daryanti Saragih I., Yang Y.P., Saragih I.S., Batubara S.O., Lin C.J. (2022). Effects of resistance bands exercise for frail older adults: a systematic review and meta-analysis of randomised controlled studies. J Clin Nurs.

[29] Sun X., Liu W., Gao Y. (2023). Comparative effectiveness of non-pharmacological interventions for frailty: a systematic review and network meta-analysis. Age Ageing.

[30] Pazan F., Petrovic M., Cherubini A. (2021). Current evidence on the impact of medication optimization or pharmacological interventions on frailty or aspects of frailty: a systematic review of randomized controlled trials. Eur J Clin Pharmacol.

[31] Li P.S., Hsieh C.J., Tallutondok E.B., Peng H.J. (2022). The dose-response efficacy of physical training on frailty status and physical performance in community-dwelling elderly: a systematic review and meta-analysis of randomized controlled trials. Healthcare (Basel).

[32] Kasa A.S., Drury P., Traynor V. (2023). The effectiveness of nurse-led interventions to manage frailty in community-dwelling older people: a systematic review. Syst Rev.

[33] Wan X., Shen J., He G. (2022). Effects of traditional Chinese exercises on frailty, quality of life, and physical function on frail and pre-frail older people: a systematic review and meta-analysis. J Frailty Aging.

[34] Labra C., Guimaraes-Pinheiro C., Maseda A., Lorenzo T., Millán-Calenti J.C. (2015). Effects of physical exercise interventions in frail older adults: a systematic review of randomized controlled trials. BMC Geriatr.

[35] Veninšek G., Gabrovec B. (2018). Management of frailty at individual level - Clinical Management: systematic literature Review. Zdr Varst.

[36] Artaza-Artabe I., Sáez-López P., Sánchez-Hernández N., Fernández-Gutierrez N., Malafarina V. (2016). The relationship between nutrition and frailty: effects of protein intake, nutritional supplementation, vitamin D and exercise on muscle metabolism in the elderly. A systematic review. Maturitas.

[37] Negm A.M., Kennedy C.C., Thabane L. (2019). Management of frailty: a systematic review and network meta-analysis of randomized controlled trials. J Am Med Dir Assoc.

[38] Esfandiari E., Miller W.C., Ashe M.C. (2021). The effect of telehealth interventions on function and quality of life for older adults with pre-frailty or frailty: a systematic review and meta-analysis. J Appl Gerontol.

[39] Apóstolo J., Cooke R., Bobrowicz-Campos E. (2018). Effectiveness of interventions to prevent pre-frailty and frailty progression in older adults: a systematic review. JBI Database Syst Rev Implement Rep.

[40] Barker S.L., Maguire N., Gearing R.E. (2021). Community-engaged healthcare model for currently under-served individuals involved in the healthcare system. SSM Popul Health.

[41] Kenny A.M., Kleppinger A., Annis K. (2010). Effects of transdermal testosterone on bone and muscle in older men with low bioavailable testosterone levels, low bone mass, and physical frailty. J Am Geriatr Soc.

[42] Arakelyan S., Mikula-Noble N., Ho L. (2023). Effectiveness of holistic assessment-based interventions for adults with multiple long-term conditions and frailty: an umbrella review of systematic reviews. Lancet Healthy Longev.

[43] Latham N.K., Bennett D.A., Stretton C.M., Anderson C.S. (2004). Systematic review of progressive resistance strength training in older adults. J Gerontol A Biol Sci Med Sci.

[44] Adekpedjou R., Léon P., Dewidar O. (2023). Effectiveness of interventions to address different types of vulnerabilities in community-dwelling older adults: an umbrella review. Campbell Syst Rev.

[45] Liu C., Xu H., Chen L., Zhu M. (2022). Exercise and nutritional intervention for physical function of the prefrail: a systematic review and meta-analysis. J Am Med Dir Assoc.

[46] Jadczak A.D., Makwana N., Luscombe-Marsh N., Visvanathan R., Schultz T.J. (2018). Effectiveness of exercise interventions on physical function in community-dwelling frail older people: an umbrella review of systematic reviews. JBI Database Syst Rev Implement Rep.

