# Supplementary material for: Comprehensive geriatric assessment and primary care based interventions for managing frailty in older adults: An evidence map
Source: J Frailty Aging. 2025 Dec 12;15(1):100104. doi: 10.1016/j.tjfa.2025.100104 (PMC12757642; doi:10.1016/j.tjfa.2025.100104)
Supplement: Supplementary file 1 [file mmc1.docx]

# Supplementary File

## 1. Supplementary Table 1: Search Strategy

| **Database** | **Filter** | **Search Details** | **Results** | **Date** |
| --- | --- | --- | --- | --- |
| PubMed | Meta-Analysis, Systematic Review | ("frail*"[Title/Abstract] OR "Frailty"[Title/Abstract] OR "Frailty"[MeSH Major Topic])) | 2379 | 04/04/24 |
| Embase | Meta-Analysis, Systematic Review | frail*:ti,ab AND ([cochrane review]/lim OR [systematic review]/lim OR [meta analysis]/lim) | 1046 | 04/04/24 |
| CINAHL | Meta-Analysis, Systematic Review | ((TI frail* OR AB frail*) OR (TI Frailty OR AB Frailty) OR (MM Frailty+)) | 468 | 04/04/24 |
| PsycINFO | Meta-Analysis, Systematic Review | (frail*.ti,ab. OR Frailty.ti,ab. OR exp *Frailty/) | 54 | 04/04/24 |
| CENTRAL | Meta-Analysis, Systematic Review | TITLE-ABSTRACT(frail*) OR TITLE-ABSTRACT(Frailty) OR MESH-TERMS(Frailty) | 11 | 04/04/24 |

## 2. Supplementary Table 2: Systematic review rating according to AMSTAR-2 criteria.

| **Sl. No.** | **Study** | **1** | **2** | **3** | **4** | **5** | **6** | **7** | **8** | **9** | **10** | **11** | **12** | **13** | **14** | **15** | **16** |
| --- | --- | --- | --- | --- | --- | --- | --- | --- | --- | --- | --- | --- | --- | --- | --- | --- | --- |
| 1 | deLabra 2015 | Y | Y | Y | Y | Y | Y | Y | Y | N | Y | N | N | N | Y | N | Y |
| 2 | Li 2022 | Y | Y | Y | Y | Y | Y | Y | Y | Y | Y | Y | Y | Y | Y | N | N |
| 3 | Dedeyne 2017 | Y | Y | Y | Y | Y | Y | Y | Y | Y | Y | N | N | Y | Y | Y | Y |
| 4 | Han 2023 | Y | Y | Y | Y | Y | Y | Y | Y | Y | Y | N | N | Y | Y | Y | Y |
| 5 | Macdonald 2020 | Y | Y | Y | Y | Y | Y | Y | Y | Y | Y | Y | Y | Y | Y | Y | Y |
| 6 | VeninÅ¡ek 2018 | N | Y | Y | Y | Y | Y | Y | Y | N | Y | N | N | N | N | N | Y |
| 7 | Artaza-Artabe 2016 | Y | N | Y | Y | Y | Y | Y | Y | N | Y | N | N | N | N | N | Y |
| 8 | Moraes 2021 | Y | Y | Y | Y | Y | Y | Y | Y | Y | Y | Y | Y | Y | Y | Y | Y |
| 9 | Kasa 2023 | Y | N | Y | Y | N | Y | Y | Y | Y | Y | N | N | Y | N | N | Y |
| 10 | Negm 2019 | Y | Y | Y | Y | Y | Y | N | Y | Y | N | Y | Y | Y | N | N | N |
| 11 | Esfandiari 2021 | Y | Y | Y | Y | Y | Y | Y | Y | Y | Y | N | N | N | Y | Y | Y |
| 12 | DaryantiSaragih 2022 | Y | Y | Y | Y | Y | Y | Y | Y | Y | Y | Y | Y | Y | Y | Y | N |
| 13 | Wan 2022 | N | N | Y | Y | N | Y | Y | Y | Y | Y | Y | Y | Y | Y | Y | Y |
| 14 | Travers 2019 | N | Y | Y | Y | N | N | Y | Y | N | Y | N | N | N | N | N | Y |
| 15 | Pazan 2021 | Y | Y | Y | Y | Y | N | Y | Y | Y | N | N | N | Y | N | N | Y |
| 16 | Sun 2023 | Y | Y | Y | Y | Y | Y | Y | Y | Y | Y | Y | Y | Y | Y | Y | Y |
| 17 | Apóstolo 2018 | Y | Y | Y | Y | Y | Y | Y | Y | N | N | N | N | N | N | N | Y |
